# Development and Process Evaluation of a Complex Intervention for Improving Nutrition among Hospitalised Patients: A Mixed Methods Study

**DOI:** 10.3390/healthcare7020079

**Published:** 2019-06-24

**Authors:** Shelley Roberts, Laurie Grealish, Lauren T. Williams, Zane Hopper, Julie Jenkins, Alan Spencer, Andrea P. Marshall

**Affiliations:** 1 School of Allied Health Sciences, Gold Coast Campus, Griffith University, Southport, QLD 4222, Australia; lauren.williams@griffith.edu.au; 2 Gold Coast Hospital and Health Service, 1 Hospital Blvd, Southport, QLD 4215, Australia; l.grealish@griffith.edu.au (L.G.); zane.hopper@health.qld.gov.au (Z.H.); julie.jenkins@health.qld.gov.au (J.J.); alan.spencer@health.qld.gov.au (A.S.); a.marshall@griffith.edu.au (A.P.M.); 3 Menzies Health Institute Queensland, Gold Coast Campus, Griffith University, Southport, QLD 4222, Australia; 4 School of Nursing and Midwifery, Gold Coast Campus, Griffith University, Southport, QLD 4222, Australia

**Keywords:** complex interventions, hospital-acquired malnutrition, integrated knowledge translation, process evaluation, staff perspectives and experiences, safety and culture

## Abstract

Hospital-acquired malnutrition is a significant issue with complex aetiology, hence nutrition interventions must be multifaceted and context-specific. This paper describes the development, implementation and process evaluation of a complex intervention for improving nutrition among medical patients in an Australian hospital. An integrated knowledge translation (iKT) approach was used for intervention development, informed by previous research. Intervention strategies targeted patients (via a nutrition intake monitoring system); staff (discipline-specific training targeting identified barriers); and the organisation (foodservice system changes). A process evaluation was conducted parallel to implementation assessing reach, dose, fidelity and staff responses to the intervention using a mixed-methods design (quantitative and qualitative approaches). Staff-level interventions had high fidelity and broad reach (61% nurses, 93% foodservice staff and all medical staff received training). Patient and organisation interventions were implemented effectively, but due to staffing issues, only reached around 60% of patients. Staff found all intervention strategies acceptable with benefits to practice. This study found an iKT approach useful for designing a nutrition intervention that was context-specific, feasible and acceptable to staff. This was likely due to engagement of multiple disciplines, identifying and targeting specific areas in need of improvement, and giving staff frequent opportunities to contribute to intervention development/implementation.

## 1. Introduction

Malnutrition is a significant issue that health organisations, clinicians and researchers have attempted to address for decades [1,2]. Despite prolonged efforts, malnutrition continues to affect up to 50% of hospitalised patients [3], resulting in poor outcomes for both patients and hospitals [4,5]. Malnutrition may be pre-existing (i.e., patients are malnourished upon admission) or hospital-acquired (i.e., patients become malnourished during hospitalisation). Approximately 5400 episodes of hospital-acquired malnutrition occur annually in Australian hospitals [6] with financial consequences, now that health services receive funding penalties for hospital-acquired complications such as malnutrition [7]. The aetiology of malnutrition in hospitals is complex and multifactorial, and problems may be patient, staff or care-related, or arise from organisational issues [1,8]. The extent of contributing factors is likely to differ across hospitals and even wards; hence, interventions targeting improved nutrition should be multifaceted and tailored to specific contexts and populations.

Developing and implementing health interventions that are feasible, acceptable and effective in the hospital setting is challenging. This is especially the case with nutrition interventions, where previous research has shown to be either effective but not practical or sustainable (e.g., blanket oral nutrition supplementation, shown to be effective in randomised control trials but not translated into practice [9]); or feasible but not effective (e.g., protected meal times [10]). To optimise the chance of interventions being successful, researchers can use a co-development approach known as integrated knowledge translation (iKT) [11]. This involves engaging knowledge end-users (e.g., health care staff and consumers) in the entire research process, so the knowledge generated is relevant and appropriate, and hence more likely to be acceptable to end-users. 

Our team used this approach to develop a multifaceted, tailored intervention to improve nutrition care, delivery and intakes among patients in an acute medical setting. Understanding the processes underpinning development and implementation of interventions is important when evaluating its outcomes to give insight into what worked, for whom and under what conditions. The medical research council (MRC) of the United Kingdom recommends conducting process evaluations for complex interventions for more meaningful understanding of its effects [12,13]. The aim of this study is to describe the development and process evaluation of a complex intervention for improving nutrition among patients in a hospital acute medical ward setting.

## 2. Methods

### 2.1. Study Overview

**Figure 1:**
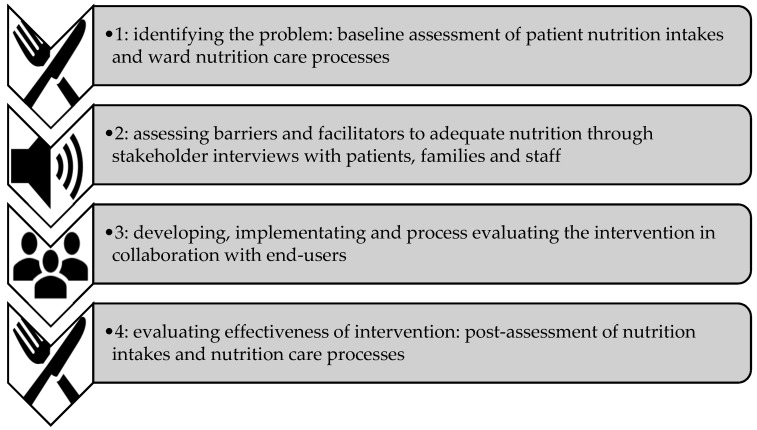
Four-step process used to develop, implement and evaluate the intervention.

This program of research used an iKT approach [11] to develop, implement and evaluate a targeted, multifaceted intervention aimed at improving nutrition intakes, care and delivery in an acute medical setting. The study was informed by principles of person-centred care [14] and guided by the knowledge to action (K2A) cycle [15]. It included four phases consistent with the K2A and French’s four-step approach [16] (Figure 1). This paper describes Phase 3 of the research.

### 2.2. Study Design

A process evaluation was conducted parallel to the implementation of the intervention, following MRC guidance for process evaluation of complex interventions [13]. The process evaluation included assessment of implementation processes (i.e., what is delivered and how; reach, dose and fidelity of delivery); and mechanisms of impact (i.e., participant responses to/interactions with the intervention). The study, consisting of mixed-methods design, used quantitative and qualitative approaches to give meaningful insights into the development, implementation and effects of the intervention. Ethical approval was gained through the relevant hospital and university Human Research Ethics Committees (HREC/14/QHC/7; NRS/22/14/HREC).

### 2.3. Setting and Sample

The study was conducted in the acute medical unit (AMU) at a 364-bed public metropolitan teaching hospital in southeast Queensland. The AMU is a 28-bed ward with a 1:4 to 1:6 nurse-to-patient ratio. Participants were patients and family members receiving care on the ward and staff providing care/services to the ward (e.g., doctors, registered and enrolled nurses, allied health professionals (dietitians and speech pathologists), and foodservice staff).

### 2.4. Research Co-Development with Knowledge End-Users

The research team engaged knowledge end-users in three ways: (1) including end-users on the study team; (2) consulting with a nutrition reference group developed specifically for the study; and (3) engaging in regular group discussions with staff on the study ward (see Table 1). The research team was multidisciplinary, including clinicians, researchers and students from nursing and nutrition backgrounds. Importantly, the team contained key staff who held positions of influence on the AMU, including the clinical nurse facilitator and the ward dietitian; or at higher levels within the organisation, such as the director of nutrition and food services and foodservices dietitian. A nutrition reference group was formed, with hospital representatives from nursing, nutrition and dietetics, speech pathology, and foodservices; a consumer representative; and selected members of the study team. Regular consultation was performed with this group in study design, data collection and analysis, and dissemination of findings from each study phase. The team liaised closely with the reference group during intervention development, implementation and evaluation. Finally, the team arranged regular group sessions with staff on the study ward to feedback findings from each study phase and facilitate group discussions about potential intervention strategies. 

### 2.5. Intervention Development

Findings from Phase 1 (baseline meal audits) and Phase 2 (stakeholder interviews) informed the intervention design, in conjunction with theory/evidence from the literature. Baseline meal audits highlighted that nutritional intakes among patients on the AMU were poor and that several factors affected nutrition at patient, staff and organisational levels. Interviews with patients, families and staff provided more detail on why and how these complex factors occurred and interacted, and how they might be addressed. Interview findings are reported in detail elsewhere [17] but in summary, they showed: (1) disciplines often worked in isolation, reflecting a siloed approach to nutrition care; (2) nutrition care was often perceived as being of lower importance among competing clinical priorities/pressures; and (3) nurses were mainly responsible for helping patients to eat, however, an insufficient number of staff were available to assist at mealtimes.

In conjunction with the nutrition reference group, the research team brainstormed possible intervention strategies targeting the identified problem areas at patient, staff and organisation levels. Focus groups were held with nursing, foodservice, and allied health staff on the AMU to share the findings from Phases 1 and 2 and to seek feedback. In these sessions, a member of the research team presented findings from previous study phases and asked staff if the findings resonated with their experiences with nutrition care on the ward. Next, the researcher facilitated discussions around possible strategies to address the problem areas identified, allowing staff to present their ideas first and then seeking feedback on the strategies brainstormed by the research team and nutrition reference group. Notes taken in these sessions were collated and used by the study team and nutrition reference group to finalise the intervention plan. The intervention components are detailed in Table 2.

### 2.6. Data Collection

Quantitative data were collected on intervention implementation (i.e., reach, dose, fidelity) during Phases 3 (implementation) and 4 (alongside post-intervention audit). For patient-level interventions, researchers recorded use of nutrition intake magnets on patient whiteboards. Staff training logs were used to evaluate intervention reach (number of staff present at each training session), dose (time spent delivering each session) and fidelity (content covered in each session) of the discipline-specific staff training. Data on organisation-level interventions (earlier breakfast delivery and use of new ‘full + hot breakfast’ diet) were collected during the post-intervention audit period. Qualitative data on end-user (nursing, allied health and foodservice staff) responses to and interactions with the intervention were collected by taking notes during informal, semi-structured individual and focus group interviews. Questions were asked using a semi-structured interview guide with questions informed by the theoretical domains framework (TDF), which is commonly used to assess barriers and facilitators to change in complex health care settings [18,19].

### 2.7. Data Analysis

Quantitative data (i.e., reach, dose and fidelity of implementation) were analysed descriptively using frequencies (number and percent) using IBM SPSS Statistics for Windows version 23.0 (Armonk, NY, USA: IBM Corp). Qualitative data were analysed using content analysis of notes taken during interviews and focus groups. 

## 3. Results

### 3.1. Implementation Processes

Patient-level implementation: nutrition intake magnets were present on patients’ whiteboards on 228 of 418 meal occasions observed (55%). They were most commonly in place at dinner (60%) and lunch (59%), whilst at breakfast only 47% of patients had magnets present. The majority of patients (64%) had green magnets, indicating adequate nutritional intake; 12% were orange (moderate risk) and 24% were red (high risk). The implementation record noted that the AMU nutrition assistant (responsible for updating magnets) was on leave for two out of the four weeks of data collection without position backfill. 

Staff-level implementation: delivery of the staff-level intervention (discipline specific staff training) in terms of fidelity, dose and reach is described in Table 3 below. 

Organisational-level implementation: the purpose-developed ‘full + hot breakfast’ diet was implemented by the foodservice dietitian prior to the Phase 4 audit. This diet was prescribed to 59% of eligible patients (i.e., those not prescribed a therapeutic diet) by nursing staff. The remaining 41% of patients were prescribed the standard ‘full diet’.

Breakfast delivery times improved from pre-intervention (delivered inconsistently, anytime between 0800–0900 h) to post-intervention (consistently delivered between 0745–0805 h).

### 3.2. Response to the Intervention

Nurses, dietitians, nutrition assistants and foodservice staff said they found the intervention acceptable; they thought it was useful to staff and helpful to patients. Their detailed feedback provides insights into possible causal mechanisms for why the intervention may have worked.

Nutrition intake magnets: the nutrition intake magnets were spoken about frequently. Staff described how the magnets prompted them to change their practice in some way, and noted that they helped indicate patients who were at risk or needed help. For example, foodservice staff described how they spent extra time assisting patients with meal set up, made an effort to communicate poor intakes to nursing staff, or left items for patients to eat later when collecting trays if they noticed a red or orange magnet. Nutrition staff thought the magnets were useful to doctors as a reminder of how they could engage in patients’ nutrition. The dietitian thought the magnets could feasibly be continued as a part of usual practice on the ward. Nurses agreed, but suggested some changes to the magnet system, such as using a single magnet with two sides (red for inadequate intake and green for adequate intake), rather than having three separate magnets (red, orange and green) to simplify the process.

‘Full + hot breakfast diet’: access to this new diet was considered by most staff to be an acceptable intervention. Foodservice and nutrition staff thought it would result in benefits such as reduced food wastage and a more patient-centred approach to nutrition. This diet was seen to reduce menu fatigue, with four hot options available each day; and patients had the ability to order hot items only if they wanted to (rather than being ‘prescribed’ foods or supplements as per usual practice), thus limiting food wastage. The nutrition assistant indicated this diet was so useful it was since being applied to patients all around the hospital (i.e., outside of the AMU) even after the study finished.

Nursing staff engagement: when asked why they thought the intervention worked, nutrition staff highlighted the engagement of nursing staff on the AMU played an integral role in the implementation and uptake of the intervention. The leadership from nurses in authoritative positions (i.e., nurse unit manager and clinical nurse facilitator) and support from nurses who were key influencers were thought to positively affect the responses and behaviour of all nurses on the ward. The dietitian said AMU nurses already had a good knowledge and understanding of the importance of nutrition and described them to be nutrition-focused, which she thought was another reason as to why they responded well to the intervention. The nutrition assistant described high nurse engagement in the intervention; for example, some nurses helped to update patients’ nutrition intake magnets even though this was proposed as the nutrition assistant’s role. Interestingly, whilst other professions highlighted that nurses played a central role in the success of the intervention, nurses themselves did not acknowledge this. Even after being shown the study findings, some nurses showed apprehension in believing the changes in nutrition intakes were a result of the intervention and sought alternate explanations. Several nurses proposed other factors that they thought might have affected the results; however, the researcher facilitating focus groups was able to explain that these factors were not at play or had already been accounted for. For example, some nurses thought the post-intervention data collection may have occurred when more students were present on the ward, so more patients may have received feeding assistance. However, pre- and post-intervention data collection periods were done at the same time of the year (February–March) one year apart, with similar student numbers on the ward in both time periods. After these explanations, nurses were more accepting of the results. Some nurses suggested the regular presence of researchers on the ward (e.g., study investigators during stakeholder meetings and research assistants conducting data collection) helped to raise the profile of nutrition in practice. 

Medical staff engagement: the dietitian found that having a medical consultant supportive of the intervention from the outset had a large impact on the receptiveness of the rest of the medical team to the intervention. She described how one consultant, who already prioritised nutrition as an important part of patient care, made an effort to communicate the medical staff training aspect of the intervention (i.e., the need for doctors to talk to patients about nutrition during ward rounds) to the rest of their team. Some nurses reported that they had heard doctors asking patients about nutrition (food intake, weight loss, etc.) during ward rounds, and said one team in particular incorporated this into their rounds and their medical notes in the electronic medical record. 

An unexpected outcome of the intervention was that tensions between foodservice and nursing staff described in Phase 2 during pre-intervention staff interviews were not apparent in post-intervention focus groups. When asked what (if anything) had changed in this respect (e.g., around communication issues raised previously), most staff said nothing had changed. When asked/prompted about the issues raised previously, both nursing and foodservice staff said these were not really an issue.

Clinical effectiveness of the intervention (i.e., outcomes of pre/post implementation evaluation) is reported elsewhere [20]. 

## 4. Discussion

In this mixed method study, the development, implementation and process evaluation of a tailored, multifaceted intervention for improving nutrition care, delivery and intakes among patients in an acute medical setting was described. This paper highlights the importance of an iKT approach (i.e., involving end-users) in the development, implementation and evaluation of complex interventions. Changing practice is widely acknowledged to be a difficult process. Knowledge translation approaches make practice change more achievable by partnering with knowledge end-users throughout the entire research process and co-developing interventions and implementation strategies [11]. The use of this approach was likely a major factor contributing to the success of the intervention, which staff echoed in post-intervention interviews. Key stakeholders were involved throughout the entire study at three levels: as members of the study team; as members of a nutrition reference group; and in frequent focus groups with ward staff. In previous research, a similar approach was used to develop and implement an intervention designed to improve malnutrition screening at a Canadian hospital [21]. The use of nutrition champions, supported by KT facilitators and an action planning process, resulted in an increase in timely assessments of patients at risk of malnutrition, as well as promoting innovations in nutrition care [21]. The success lay in the ward-based nutrition champions and consideration of barriers and facilitators at micro (ward), meso (organisation) and macro (healthcare system) levels [21]. Similarly, our study assessed issues and targeted intervention strategies at multiple levels (patient, ward/staff and organisational levels) and utilised ward-based staff as facilitators/champions. Many staff, including the ward dietitian, attributed much of the intervention’s success to the active role of influential staff on the ward (e.g., clinical nurse facilitator) and ‘owning’ of the intervention by ward nurses. The focus groups with ward nurses were particularly important, as they not only provided information for intervention development, but also acted as part of the intervention itself by: (a) increasing staff awareness about nutrition (specific to their ward and patients); and (b) giving staff ownership of the intervention by engaging them in its development (e.g., by providing ideas based on their practical experiences).

Intervention reach for staff training was high (60% of nurses; 100% of RMOs and >90% of foodservice staff received training), likely due to influential staff being on the study team (clinical nurse facilitator, foodservices dietitian, AMU dietitian) or supportive of the study (nurse unit manager, foodservice manager, a medical consultant). Fidelity was 100% across all staff groups (all elements of training delivered as planned) and dose was similar across staff in each group. This was reflected in other process outcomes, such as patient-level interventions; 59% of patients eligible for the ‘full + hot breakfast diet’ were allocated it by nurses, and 55% of patients had nutrition intake magnets in place during the post-intervention audit. It was noted that the nutrition assistant, who was responsible for updating patients’ nutrition intake magnets, was on leave for two of the four weeks that the audit took place, and her position was not backfilled. Hence, the finding that only about half the patients had their magnets updated daily is not surprising. We did note that some nurses took on this responsibility in some instances; however, temporary/casual nursing staff likely did not receive the training so they would not know what the magnets meant. Several nurses suggested using a single magnet with two sides rather than having three separate magnets, which they thought would simplify the process in several ways. Firstly, they highlighted that the orange magnet was redundant as patients either ate well or ate poorly, and the system should reflect this. Secondly, by using just one double-sided magnet (green for eating well; red for eating poorly), nurses could keep whiteboards updated more easily as they would not have to go looking for the correctly coloured magnet. The nutrition assistant kept the coloured magnets in a box, and not all nurses knew where it was kept; whereas a double-sided magnet would always be present on each patient’s whiteboard. These suggestions were important for future tailoring of the intervention. Finally, the organisational-wide strategy (moving breakfast to an earlier time) was effective, with breakfast consistently being delivered at 0800 h post-intervention, compared to inconsistent delivery between 0800–0900 h pre-intervention. This change was important, as it enabled the ward to meet one of Queensland Health Statewide Foodservices Network’s key performance indicators of no longer than 13 h between the last meal service and the next day’s first meal service [22]. However, the feasibility of implementing this change across the hospital was challenging due to foodservice staff rostering, as shifts had to be rostered earlier to accommodate this.

Responses by staff to the intervention were mostly positive, and several discussed how they thought it worked to improve nutrition care, delivery and intakes (i.e., suggested mechanisms of action). Many of their perceptions aligned with the TDF, which is often used to explain human behaviour in complex clinical environments [18]. For example, staff found the ‘full + hot breakfast’ diet highly acceptable, as they saw benefits to it (e.g., reduced menu fatigue and food wastage, more patient-centred), which is consistent with the ‘beliefs about consequences’ domain. 

Nurses’ high engagement in the intervention correlated with several theoretical domains. Firstly, nutrition staff thought nurses responded well to the intervention as they generally had a good knowledge and understanding of the importance of nutrition and described the ward to be ‘nutrition-focused’; aligning with domains ‘knowledge’, ‘skills’ and ‘environmental context and resources’ (specifically, ward culture). Also, nurses seemed to generally take ownership for nutrition and saw it as their role, which translated into some nurses taking responsibility for intervention strategies (e.g., updating nutrition intake magnets); consistent with the ‘social/professional role and identity’ domain. Finally, the leadership, support and influence of nurses in authoritative roles (e.g., nurse unit manager, clinical facilitator) were seen to play a large role in engaging all ward nurses and ultimately the intervention’s success, consistent with previous literature [23]. This aligns with the TDF’s ‘social influences’ domain, whereby interpersonal processes cause individuals to change their thoughts or behaviours.

Several staff said the nutrition intake magnet system prompted them to change their practice or behaviour. For example, nurses said it helped them identify at-risk patients, foodservice staff said they spent more time assisting patients with meal set-up and communicate with nurses, and the dietitian thought it helped to remind doctors to discuss nutrition. This aligns with the ‘memory, attention and decision processes’ domain, which involves retaining information, focusing selectively on aspects of the environment and making clinical decisions [18]. This may be of advantage in busy clinical environments when staff may be cognitively overloaded or tired. The nutrition intake magnets acted as a reminder to staff to undertake the nutrition care tasks they received training on (i.e., those outlined in Table 1) and is an example of a successful discrete implementation strategy [24]. 

Processes relating to the iKT approach used may have had indirect effects on the success of the intervention. For example, the regular presence of nutrition researchers on the ward and regular contact between researchers and ward staff could have increased staff awareness and increased the profile of nutrition. The fact that the ward dietitian, nurse unit manager and nurse clinical facilitator were involved in the study may have increased nurses’ acceptance of it. Regular updates provided to ward staff (such as presenting findings from each study phase and facilitating discussions around these) may have acted as an audit and feedback type mechanism, whereby staff were provided with data specific to their ward and patients, highlighting areas in need of improvement [25]. Finally, as ward staff were regularly consulted with and contributed to intervention design, they likely had more ownership of it, which may have increased acceptance of the practice changes required.

This study has several limitations. Firstly, the intervention was evaluated using a pre/post design (findings will be reported elsewhere), which was most feasible for the co-development approach. Secondly, this study was conducted in one medical ward, at one Australian hospital, so the findings cannot be generalised. However, this detailed example of how to use an iKT approach to develop, implement and evaluate a multifaceted intervention targeting improved nutrition among hospitalised patients provides an implementation model for testing in other sites. Finally, due to the limited study timeframe, we were unable to assess intervention sustainability. 

## 5. Conclusions

This study used an iKT approach to develop and implement a complex, tailored intervention aiming to improve nutrition care, delivery and intakes among patients in an acute medical ward. This paper described the intervention co-development process (with end-users) and presented an evaluation of the processes underpinning implementation in a real-world clinical setting. Quality of intervention delivery (reach, dose, fidelity) was high, which is most likely attributed to the strong stakeholder engagement. In particular, involvement of nurses in authoritative positions on the ward (such as the clinical facilitator) seemed to increase acceptance and use of intervention strategies by ward nurses. The engagement of multiple disciplines, the identification and targeting of specific areas in need of improvement in that ward, and providing staff with frequent opportunities to contribute to intervention development and implementation were also important. Whilst developing the intervention was time and resource intensive, it ultimately resulted in positive outcomes. Hence, sharing our learnings is important for advancing implementation science in the field of nutrition by providing an example of how an iKT approach can be used to develop and implement a malnutrition prevention intervention in an acute medical setting.

## Figures and Tables

**Figure 2:**
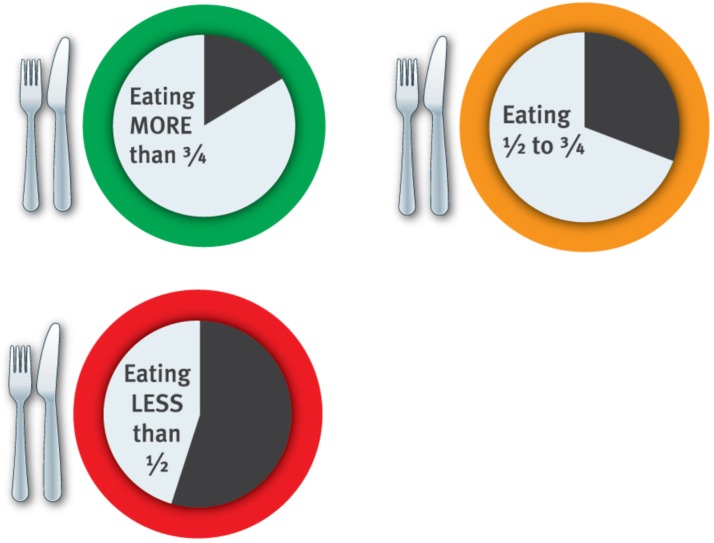
Patient nutrition intake whiteboard magnets.

**Table 1:** Engagement of knowledge end-users.

Level of Engagement	Team Members			Roles
	Academics	Clinicians	Consumers	
**Study team**	Prof of Nursing	Nursing clinical facilitator (ward)	N/A	Involved in all aspects of research, i.e.: study design; data collection and analysis; intervention development, implementation and evaluation.
	Prof of N and D			
	A/Prof of Nursing	Dietitian (ward)		
	Research Fellow (N and D)	Foodservice dietitian		
	3 × N and D Honours students	Director of nutrition and foodservices		
**Nutrition reference group**	Prof of Nursing *	Dietitian (ward)	Consumer (patient) representative	Contributed to intervention development, implementation and evaluation.
	Research Fellow (N and D) *	Foodservice dietitian		
		Speech pathologist		
		Nursing clinical facilitator (ward)		
		Manager of foodservices		
**Ward staff focus groups**	N/A	Nursing staff	N/A	Contributed to intervention development.
		Allied health		
		Foodservice staff		

N and D: nutrition and dietetics, N/A: not applicable, Prof: professor, A/Prof: associate professor, * same members of study team as described in row above.

**Table 2:** Intervention components.

Intervention Component: Description and Purpose		Rationale (Causal Assumptions)	Delivery
**Patient level intervention: nutrition intake magnets**			
Traffic-light nutrition intake magnet system (A8 size), displayed on patient bedside whiteboards to indicate if patient is eating well (green), poorly (orange; eating <75% meals) or very poorly (red; eating <50% meals), see Figure 2. This visible tool helped staff identify patients at greatest nutritional risk (in order to prioritise care), acted as a reminder to staff of their discipline-specific training and generated conversations about nutrition between staff and patients.		Phases 1 and 2 indicated the need for a whole team approach to nutrition (as nutrition care was siloed). Nutrition was not always of high importance to some staff groups when planning patient care, and staff needed a way to identify high-risk patients who required additional care/support to achieve recommended intakes.	AMU nutrition assistant ^a^
Staff level intervention: discipline-specific training			
Discipline-specific training targeted at nurses, doctors and foodservice staff. All groups received generic content on malnutrition; an overview of Phase 1 and 2 study findings (including feedback on patient intakes); and introduction to nutrition intake magnet system. This was to increase staff awareness of nutrition and of the study, facilitate stakeholder engagement through communicating study findings, and to familiarise staff with the intervention.		Data from Phases 1 and 2 indicated that each staff group could play an important role in patients’ nutrition care. Theory (iKT) suggests dissemination of findings to and engagement with end-users is important for intervention uptake.	
Nurses	Nurses were trained on improving meal access and uptake, through: - Preparation for meal delivery: ensure tables are clear and that foodservice staff place trays within patient reach - Meal set up and feeding assistance: prioritise using magnet system and ask other staff (i.e., allied health assistants) for help when possible - Provide patients with encouragement to eat and speak about meals in a positive way - Limit unnecessary interruptions during mealtimes and use unavoidable interruptions as an opportunity to assist or encourage patients to eat - Allocate patients a ‘full + hot breakfast diet’ (see below)	Phases 1 and 2 indicated: - Meal access (placing meals within patient reach, meal set up, feeding assistance) was an issue and nurses and foodservice staff both had roles to play - How nurses talked about hospital food in front of patients (i.e., making negative comments) influenced what patients thought of meals - Mealtime interruptions could positively or negatively impact on patient intakes	AMU clinical nurse facilitator
Doctors	Doctors were encouraged to consider nutrition and discuss it in daily ward rounds, focusing on patients eating poorly (as indicated by magnets): - Try not to interrupt meals with ward rounds, tests or procedures - Ask questions like: “How is your appetite? How much have you been eating? Have you lost any weight recently?” during ward rounds - Ask patients about any barriers to eating (e.g., symptoms like nausea/vomiting, chewing/swallowing problems etc.) - Remind patients of the importance of eating enough whilst in hospital to facilitate recovery and discharge - Provide encouragement to patients to eat	Phase 1 indicated that mealtime interruptions by doctors’ ward rounds negatively impacted on patient intakes. Previous studies and findings from Phase 2 showed patients highly esteem what doctors say about nutrition; hence if doctors regularly ask patients about their appetite, intake, weight, or provide encouragement to eat, patients perceive this as important and will be more likely to actively contribute to their nutrition in hospital.	AMU dietitian
Foodservice staff	Foodservice staff were trained (on meal delivery) to: - Introduce themselves, address patient by name and tell them that they are here to give them their meal - Ensure patient’s table is in a suitable position over their lap and meal tray is placed within reach and communicate with nurses if there are items (i.e., medical equipment) on table that need moving - Ask patient if they need assistance opening packets or finding cutlery And on meal collection (if patient had not eaten much): - Check patient has been able to access/open foods and drinks on tray - Ask why they have not had anything to eat and communicate to nurse - Ask if they would like to keep something for later - Leave tray until last to collect if patient is still eating or eating slowly	Phases 1 and 2 indicated: - The manner of foodservice staff at meal delivery affected patients’ mood and perceptions of the food; - Meal access was an issue, with staff sometimes leaving trays on tables out of patients’ reach; - Some patients required limited assistance (e.g., to open packets) that could be given by foodservice staff upon tray delivery; - Some trays were taken before patients had finished eating.	Foodservice training officer and foodservice dietitian
Organisational level intervention: foodservice strategies			
Policies and procedures were changed to maximise patient intake at breakfast by: - Developing a ‘full diet + hot breakfast’ diet. This consisted of a standard ‘full’ diet with the addition of a high energy/high protein hot breakfast option (e.g., eggs, sausages or baked beans). Patients could choose from two hot breakfast items per day in addition to their standard ‘full diet’ ^b^ - Changing timing of breakfast meal (from between 0800–0900 h to 0800 h)		Previous literature and Phases 1 and 2 data indicated patients ate best at the breakfast meal, due to better appetite and more acceptable foods; however, breakfast provided less energy and protein than lunch and dinner. In Phase 1, breakfast was the most frequently interrupted meal due to its late delivery (between 0800–0900 h) clashing with doctors, allied health and nursing rounds ^d^	See below ^c^

^a^ Nutrition Assistant (NA) already conducted daily lunch audits for all patients on AMU. Data from Phase 1 showed lunch intake was a good indicator of total daily intake, so it was decided among the team and ward stakeholders that the NA would update magnets daily during this lunch audit. ^b^ On admission, patients are allocated a ‘full’ diet unless another therapeutic diet is indicated (i.e., they have dysphagia and require a texture modified diet, or they have diabetes and require a diabetic diet). As nurses allocate patients’ diets on admission, they were trained to allocate patients the ‘full + hot breakfast’ diet as part of this intervention. ^c^ The study team, key stakeholders (nurse clinical facilitator and foodservice dietitian) and foodservice department worked together to change breakfast timing. The clinical nurse facilitator provided informal training to nurses on the ward around allocation of the new diet. ^d^ The extended period of fasting (14–15 h) between dinner and breakfast the following morning was also an issue and not in line with guidelines.

**Table 3:** Delivery of staff training.

Aspect	Nurses	Doctors	Foodservice Staff
Fidelity	All components delivered	All components delivered	All components delivered
Dose	5 × 10-min training sessions delivered at daily safety scrums; and 6 × 3–4-min informal training sessions with individual nurses	2 × 10-min informal training sessions delivered to doctors at a time of convenience (i.e., in meeting rooms)	Formal training session (10-min oral presentation) to most staff, or informal small group/individual training with some staff
Reach	28 of 46 nurses (61%) employed on the ward received training	4 RMOs * and 1 consultant received training	82 of 88 staff employed at the hospital received training (93%)

RMO: resident medical officer. * Four medical teams regularly provided service to the ward, each with one RMO.
